# Correction to “P68 RNA helicase promotes invasion of glioma cells through negatively regulating DUSP5”

**DOI:** 10.1111/cas.16301

**Published:** 2024-07-29

Wang R, Bao HB, Du WZ, et al. P68 RNA helicase promotes invasion of glioma cells through negatively regulating DUSP5. *Cancer Sci*. 2019 Jan;110(1):107‐117. doi: 10.1111/cas.13858

There were errors in Figure 4A and Figure 6E in the above article.

The correct Figure 4A is shown below:

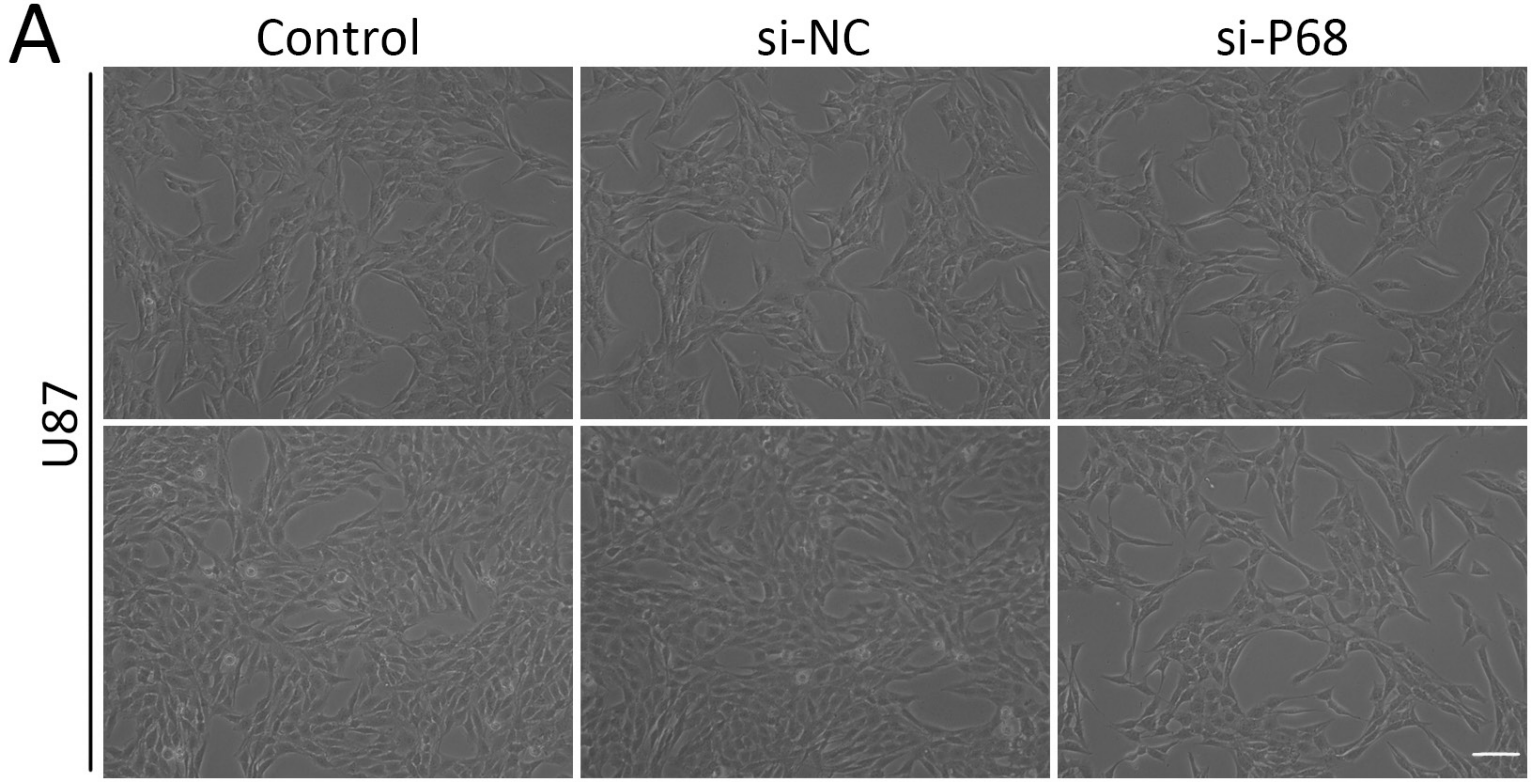

The correct Figure 6E is shown below:

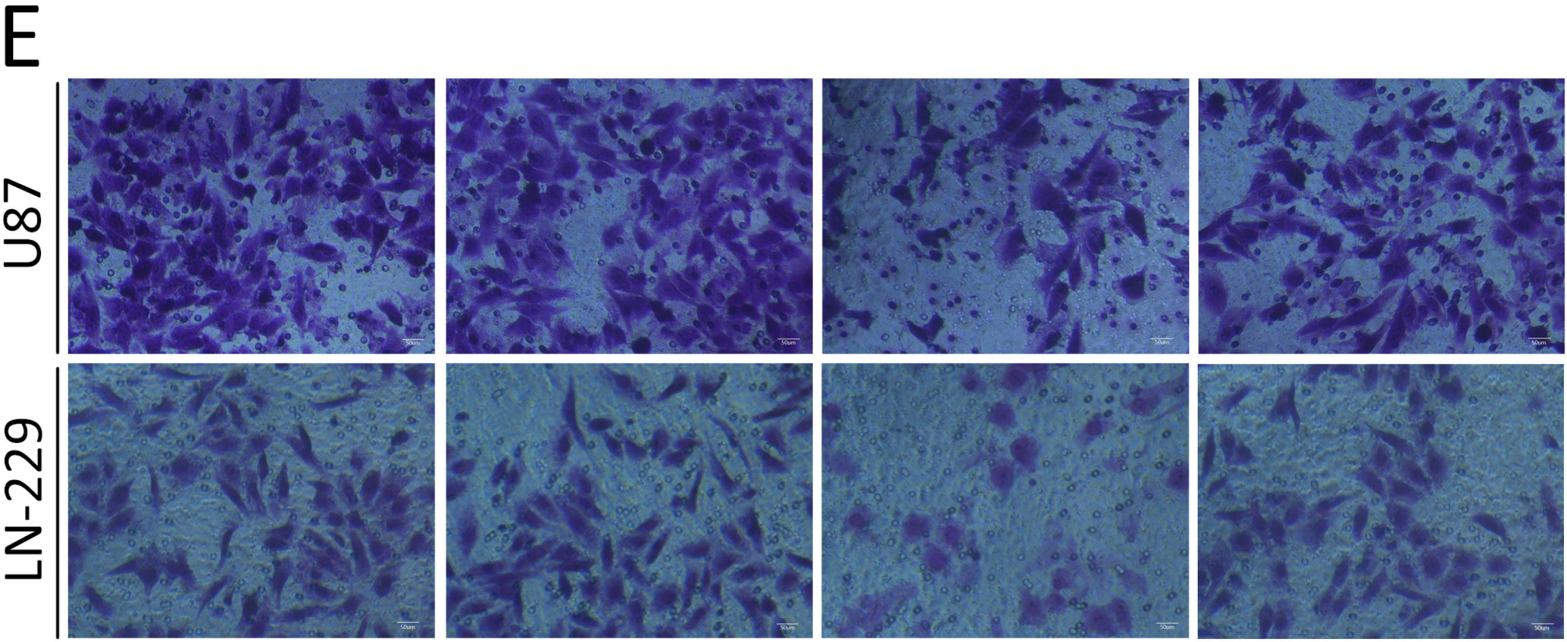

The authors apologize for the errors.

